# An Address upon the Fiftieth Memorial Celebration of the Discovery of Anæsthesia

**Published:** 1895-01

**Authors:** James E. Garretson

**Affiliations:** Philadelphia


### AN ADDRESS UPON THE FIFTIETH MEMORIAL CELEBRATION OF THE DISCOVERY OF ANÆSTHESIA.[1]

BY DR. JAMES E. GARRETSON, PHILADELPHIA.[2]

I AM to assure this great audience that I stand in its presence overwhelmed by the contrast which separates subject and speaker, and that I find words in deference only to the circumstances of the occasion that brings us together.

It is no profane comparison to suggest that naught but a sense of profanation could associate with a priest who should add words to the lifting of the Host. Does not a priest, in performance of this act, set a seal upon his lips? Does he not wrap in vestment which has been blessed the hands that are to touch the sacred symbol? The profundity of the meaning, not to say the holiness, of what seems to me to be among the greatest of God's gifts to man, anæsthesia, affects me in its contemplation, as I assume the priest

---

[1] Delivered at Philadelphia, December 11, 1894.

[2] Senior Surgeon of the Medico-Chirurgical Hospital, etc.

to be affected as he approaches the Host. The full feeling is,—be still.

Silence! and its golden meaning!

Surely,—forcing ourselves to talking and listening,—the gold of anæsthesia is silence. Silence in place of agonizing, heart-rending screams. Silence in place of cries from the lips of pitying, but helpless, by-standers. Silence in presence of torture shorn of its terror.

On an occasion, now many years back, I was wandering through the lanes and alleys of Sleepy Hollow Cemetery, in the town of Concord, Massachusetts, when, being led up a hill, I stepped over a low, much-abused hedge of arbor-vitæ, discovering a plain, low-set stone, having upon its face a single word. When at the foot of the hill, I had found myself surrounded by what would not inaptly bear description as splendid mausolea. Upon these marbles were deeply cut many names, and the records of many virtues. Neither names nor virtues had, however, significance to me. No chord was struck, no response elicited.

The word upon the low-set stone of the hill-top was Hawthorne.

The ring of a bell is its metal. The name of a man is his work. Men who have done something, either as cause or instrument. What reverberations ring out as such names are encountered! Somewhere, everywhere, is a sound. The lives of great men, the memories of the lives of great men,—left to remind us.

Horace Wells! The name does not, nor will not, still. It rings, and rings, and rings, in distinctness, albeit accordant and discordant sounds are everywhere around it.

The task of reviewing the history of anæsthesia was given to the worthy colleague who has preceded me. I am glad of it. Standing, as I feel myself to-day, overshadowed by names and memories,—memories of blighted lives, of mental wreckage, of discouragements ending in suicides,—what, but admiration of the sacrifices made; what, but desire to do homage; what else than these should or could fill a human heart on such an occasion? Here Cæsar can be praised, and Rome too.

A new good is an old gift,—not new in ages past only because channel was lacking. Electricity before chaos. An Edison the production of a nineteenth century. Euphrates, Tigris,—both water Mesopotamia; both, alike, are the Persian Gulf. Not Armenia is the source. A common under-spring is the well constituting the divine afflatus.

My perceptions view Horace Wells as Euphrates and Tigris are viewed; he, and these, and all as phenomena; not as things in themselves, but as things in other things. Whether or not this man was a meditative philosopher to be found oftenest in haunts apart from men, or whether or not he was simply a vessel capable of holding, but never trying to fill itself, I am alike without knowledge, as without desire to know. He was filled, however. The river of Lethe found in him a channel. Everywhere over the land flows the stream Nepenthe.

To change a metaphor. Is invention aught but filling a form? Is this not a matter made plain by Plato two thousand years back? Are not forms eternal?—forms of things seen yesterday, to-day, and to be seen forever? forms not yet seen? Is invention else than seeing a form and bringing it down from the sky to the uses of men? Materializing it, properly speaking. How as I grow old do I grow impressed with this: the maker of forms the all, the filler of forms, simple instrument.

It is not even slight departure from the immediate subject of the occasion to make further reference to this matter of forms, for it is not otherwise, according to my conception, that Horace Wells is to be either understood, appreciated, or called the discoverer of anæsthesia.

Was it, truthfully, Priestley to whom nitrous oxide owes its discovery? It assuredly was not to Horace Wells. Was or was not anything known of this gas before the day of our own Declaration of Independence? Or is it rather to be put thus: nitrous oxide is phenomenon deductively exposing itself to the chemist out of the droppings of camels upon desert sands; Ammon and ammonia go back to Libya.

No one has been, or is, greater than Plato. What the dung-burners did, or what Priestley has done, is not credited by him as science, but simply as dealings had with phenomena. "Science," he says, "fritters itself, where its aim is otherwise than knowledge of noumenon." Things unlike are not necessarily dissimilar. The things that lie within things are multitudinous. Who knows even yet what nitrous oxide is? Who knows even what water is? In a word, who knows what or how much anything is? Nobody.

But the scientist is the evolutionist. Possessing himself of means, he analyzes. Analysis is one, or closely one, with deduction. Science has no thought, or word, or action, outside of matter.

But forms, the true objects of science, as affirmed by Plato, constitute the invisible. The music of a musician is not his notes, the poet's inspirations are not grammar. Reality, or at least nearer approach to reality, is back of these. Notes are to be seen by anybody having eyes, and words are to be heard by anybody having ears. But what as to forms, or ghosts, as these are back of notes and words? What as to the seers of these? No forms being back of notes and words, there are no notes and words in front of forms.

Was not the ghost of anæsthesia with the camel droppings? Was it not with the dudaim, the devil's apple, of the Arab? Has it not been with alcohol since men distilled and knew this agent? Is it not with the poppy through all the ages that fields have been made red by this plant?

In 1540 the oleum vitrioli dulce was first given to the uses of men by Valerius Cordus. He had not the name ether for it, but his oil was akin with the ether of Frobensius, and with the ether of to-day. Guthrie, Liebig, and Soubeiran simultaneously discovered chloroform in 1831. Did Cordus, in 1540, see or tell anything about a ghost of anæsthesia, as this lay in his sweet oil of vitriol? With chloroform filling the bottles of druggists, in 1831, was anybody to be found who had been introduced through its use to the wonderland of Euthanasia.

Let here the idea be repeated of nobody knowing what anything is. Cadmus, beyond all men of his times, saw letters. A Shakespeare, beyond all men of his times, saw use lying with letters. How many are the expressions lying with letters not yet seen by anybody? Forecast the unwritten poetry.

Here is culmination; and here is the place of Horace Wells in history. Horace Wells saw in a room at Hartford what had never before been seen by mortal man. He saw anæsthesia. It was ages before he was born that ether was materialized, and it was before he was born that nitrous oxide was formulated, and it was when he was in no way thinking about such things that chloroform was brought forth. The seer saw anæsthesia. The sight lay, as I understand, with a hurt hand of which no complaint was made. Others, many others, saw the blooded hand. In a distant State, about the same time, I myself saw the hurt hand, but none, not one of the many others, saw anæsthesia. I think it is not to be denied that some saw a filmy halo that meant anæsthesia. There was a something, but what the something was they did not make out. What was seen was seen only to be forgotten. Truly, Sir Humphry Davy is to be credited with a sight and feeling of an elysium. Ego was differentiated for him by nitrous oxide from environment; but, while he felt and saw, it was separability that was felt and seen, not anæsthesia. His expression on coming from under the influence of nitrous oxide is familiar, "Nothing exists but thought."

Anæsthesia, the thing being truly understood, is barrier between matter which does not feel, and Ego, which is percipient. It is not matter that sees, hears, feels, touches, or tastes. Does the matter temporarily composing the cadaver of a dissecting-room see, hear, feel, touch, or taste? Percipient is away from it; there is neither seeing, hearing, feeling, touching, nor tasting by a cadaver; a flute separated from its player is wood, having no sing in it.

I am not to credit Horace Wells with sight of separability. The "Me, and the not Me," is not likely to have been seen even by his internal eye. His seership lay with a direction that people delight to call practical. Of many things, hundreds, thousands, perhaps, lying with nitrous oxide, he saw one. But what a one! Here begins his glory. Here is to continue his glory. Here, so long as pain is esteemed hurtful and absence of it pleasurable, will the name of Horace Wells be upon the lips of men.

Parallels recall obligations and glory due others. Apples have fallen since first apples began to grow and ripen. Kettles, in which water was being boiled for the evening repast, have opened their iron lips and tried for numberless centuries to say what they had to tell about locomotion. Over the earth and across the face of the heavens electricity has sought vainly until lately for a seer. The sun with his rays full of perfect pictures, brought as free gifts to men, could find no taker.

In the year 1665 a seer sitting under a tree at Woolsthorpe found himself able to hear fairly well what a falling apple had to tell about the moon staying where it belongs. Heron of Alexandria, holding his egoistic ear to the spout of a kettle, heard a story of steam and wrote it down in the shape of his æolipile. Papin saw the cylinder. Fulton saw a steamboat. Stevenson was, perhaps, the first witness of a train of cars drawn by a locomotive. Thales of Miletus got a story of electricity from a piece of amber. Daguerre, not, however, until the age of the world A.D. 1839, was able to take what the sun had to give of sun-pictures. Mozart, beginning with the use of common sense, and from this passing to the advantages lying with educated sense, dropped at the last both these and put down in form of notes what alone the flowers whispered to him,—having found out that education is not always the best teller of things that are to be heard.

Not unnecessarily to detain, was anæsthesia, as anæsthesia, known to surgery before 1844 as it became known in that year and since remains known? Not nitrous oxide, not ether, not chloroform, not rapid breathing, but anæsthesia.

Who was the man of that year? Horace Wells. Does this not settle the question?

Let, however, all deserved honor and glory associate with names of workers and experimentalists as these have enlarged application of the inspiration of the Hartford seer. Ether, chloroform, what would surgery do without them? How could the world do without them? How *did* the world do without them?

It is not necessary in this presence to enlarge beyond a sentence on the benefits of anæsthesia to humanity. Are not all here assembled doing and experiencing, each after the manner of his work, what I did and experienced only yesterday. Upon the operating-table of a hospital lay sleeping sweetly and quietly as ever baby slept a member of our fraternity. In place of an ordinary neck was a tumor that reached from chin to sternum and from ear to ear. Wherever, as it proved, reaching fingers could reach, prolongations of this mass extended themselves. Salivary glands, trachea, carotid arteries, jugular veins, pneumogastric nerves, all were more or less embraced and wrapped about; yet, while so horrible a dissection as was required to remove the mass went on, sleeping and dreaming quietly continued, nor was any consciousness had by our brother of his terrible experience until an hour later he awakened snugly tucked away in one of the most comfortable of beds, the tender hand of a godly nurse wiping away the cold sweat-drops standing threateningly upon his forehead.

Consider, in contrast, a picture familiar before the day of Wells's inspiration! A mother, her heart welling out in tears, limbs trembling so as scarcely to afford her support, helpless misery characterizing her countenance, despair striking at her with its thongs of flame, follows into a hospital operating-amphitheatre a nurse who carries her first-born, which is being brought to the table. Alas! helpless, indeed, is the mother. How more than gladly, how a thousand times more than gladly, would she lay down in place of the child! Cries of mother and child moan through the hospital, and the least sensitive feels his cheek pale. The crucial moment has come; the child is placed and held by force upon the table. The mother is torn away. For a single moment eyes of mother and child have met in parting. A loud, frightened, despairing cry from the child rings from ceiling to floor of the room. The mother drops in a heap and is carried out a raving lunatic; she raves about and curses God as being without pity or mercy.

Let a concluding picture, having relation with the first, help cover the intermediate one,—an intermediate one which extended, alas! from the days of the first surgical performance to the year of grace eighteen hundred and forty-four.

A mother brings to a hospital a child whose deformity requires the knife for its correction. Conscious of the power of anæsthesia, the surgeon talks to the parent, while all the while the little patient, pleased and inveigled by the sweet smell of chloroform, is itself anæsthetizing itself. The cutting is done. The child had a dream of roses and gardens and wide fields. The mother has placed in her arms her restored offspring. She has no tears, no words; her contact has been alone with beneficence. She is overwhelmed by the mystery she has met and passed. She says, "Our Father which art in heaven." She says and feels there is a God of pity and mercy.

Look at the name of the maker of these pictures of the new time; it reads, Horace Wells.

To what extent anæsthesia has cultivated sensibility I leave every surgeon to judge. Who, if suddenly transplanted into the olden times, being possessed of his present knowledge of anæsthetics, could handle a knife without cutting everywhere else than where it would be desirable to cut; otherwise dying shortly, out of sympathy for his patients? Could he say, "Merciful Father which art in heaven," in place of thinking, "Pitiless devil who is in hell"? Alas! how near to hopeless atheism may ignorance bring a man. Hail! that knowledge shows God and Father everywhere. Hail to all poets, to the music hearers, to the seers of forms of every kind! Let statues be stood for them in the squares. Let tablets of enduring brass mark their working-places. Let us place and hold them with the immortals.

Hail to him who has proven to be, perhaps, the greatest of the seers,—Horace Wells!

